
JOURNAL INFORMATION
==============================
NLM Title Abbreviation: Natl Sci Rev
Journal ID: Natl Sci Rev
NLM Journal ID: 101633095
Journal ID: nsr

National Science Review

ISSN: 2095-5138
EISSN: 2053-714X

ARTICLE INFORMATION
==============================
PMCID: PMC7454789
PMID: 34676081
DOI: 10.1093/nsr/nwaa168
Article ID: nwaa168
Article version: 1
Subjects: Forum, AcademicSubjects/MED00010, AcademicSubjects/SCI00010

Challenges of China's Medical Education in the coming Post-COVID-19 Era

Shao Li
Zhao Weijie
Electronic publication date: 2020 Jul 28
Electronic Location ID: nwaa168
Copyright: © The Author(s) 2020. Published by Oxford University Press on behalf of China Science Publishing & Media Ltd.
Copyright year: 2020
License: This is an Open Access article distributed under the terms of the Creative Commons Attribution License (http://creativecommons.org/licenses/by/4.0/), which permits unrestricted reuse, distribution, and reproduction in any medium, provided the original work is properly cited.
License URL: https://creativecommons.org/licenses/by/4.0/

edited-state: accepted-manuscript
article-lifecycle: PAP

==============================
**Forum**

### Challenges of China’s Medical Education in the coming Post-COVID-19 Era

Li Shao and Weijie Zhao

The year of 2020 is overshadowed by the COVID-19 pandemic. Medical workers throughout China have played critical roles in battling coronavirus and saving lives. The whole society now fully realized the significance of medical workers and many began to think about medical education in China: How can we further improve medical education for the next generation of clinicians, medical scientists, nurses, public health workers, and administrators related to medical care, so that they are well prepared to meet societal needs for medical care in the ever changing world? In this panel discussion, medical education experts from several prominent medical schools gathered to discuss the reform and future development of medical education in China.

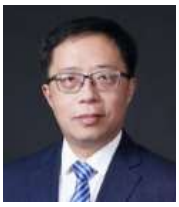

**Xiang Chen**
President of Xiangya School of Medicine, vice-President of Central South University, Changsha, China

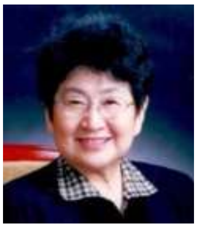

**Baorong Chi**
Professor of Norman Bethune Health Science Center of Jilin University, Changchun, China

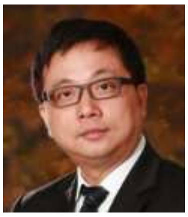

**Yiqun Hu**
Vice-President of Shanghai Jiao Tong University School of Medicine, Shanghai, China

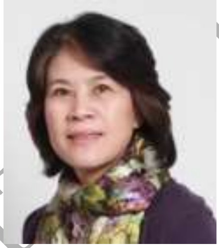

**Yang Ke**
Professor of Peking University Health Science Center, former vice-President of Peking University, Beijing, China

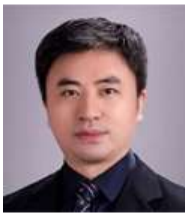

**Ming Kuang**
Vice-President of Zhongshan School of Medicine, Sun Yat-sen University, Guangzhou, China

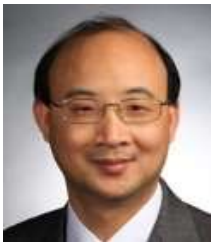

**Mengfeng Li**
President of Southern Medical University, Guangzhou, China

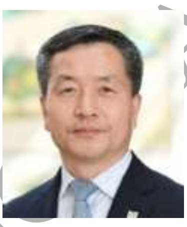

**Hongbing Shen**
President of Nanjing Medical University, Nanjing, China

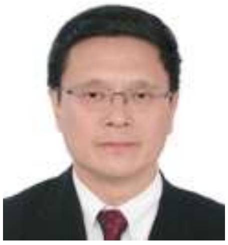

**Xuehong Wan**
Professor of West China Medical Center, vice-President of Graduate School of Sichuan University, Chengdu, China

ORIGINAL UNEDITED MANUSCRIPT

© The Author(s) 2020. Published by Oxford University Press on behalf of China Science Publishing & Media Ltd. This is an Open Access article distributed under the terms of the Creative Commons Attribution License (http://creativecommons.org/licenses/by/4.0/), which permits unrestricted reuse, distribution, and reproduction in any medium, provided the original work is properly cited.

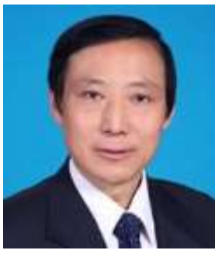

**Hong Yan**
President of Xi'an Jiaotong University Health Science Center, vice-President of Xi'an Jiaotong University, Xi'an, China

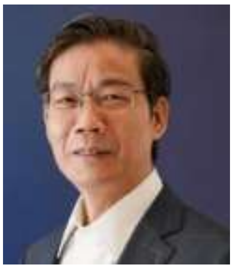

**Guoqiang Chen (Chair)**
Chancellor of Shanghai Jiao Tong University School of Medicine, vice-President of Shanghai Jiao Tong University, Shanghai, China

**GQ Chen**: The COVID-19 pandemic has been the biggest public health emergency since the founding of the People's Republic of China. The entire country was mobilized to fight the disease. Around 42 600 Chinese medical workers joined this battle. They bravely headed to the front line and saved many lives in Wuhan, Hubei province and all over China. Many heroic stories highlighted the spirit of these workers - the unyielding resolve to save lives with all their strength and love.

I believe that most people had never before better realized that, medical workers are so precious and should be fully respected, and medical science should be highly valued as a science that can save the nation. Also, further reinforcement of medical education, which forms the basis for the medical and health care system, should be put into the society's immediate agenda in the post-COVID-19 era.

Medical education in China has been greatly improved over the past decades. But in order to step up further, we should first objectively recognize the existing problems, especially those exposed during the pandemic. That is what we are going to discuss today: the problems and the possible solutions.

### The importance of preventive medicine

**GQ Chen**: The object of medicine is humans. Humans are complicated both as an animal species and as members of the civilization. So, medical science is a combination of natural science, social science and the humanities. It is the most logical one among all social sciences and the most humanistic one in all natural sciences.

Medical science includes lots of aspects, including clinical medicine, basic biomedical research, preventive medicine, nursing, medical technology, medical humanities, medical sociology, and health economics. Medical science is the entirety of all these aspects and its integrity and particularity should be respected.

ORIGINAL UNEDITED MANUSCRIPT

**BR Chi**: That' right. We should respect and protect both the integrity and particularity of medical science. The COVID-19 pandemic exposed the problem that our public health system had paid much more attention to treatment than prevention. We do not have enough public health workers. On the one hand, it is hard to cultivate qualified public health workers; on the other hand, they are not fully respected by the society.

Perhaps we should readjust the proportion of students in different disciplines of the medical school and teach more about serious infectious diseases in order to cultivate medical talents with high professional competency. And of course, the government should better support public health workers.

**HB Shen**: The entire society, not only the medical schools, tends to pay more attention to treatment than prevention, resulting in the separation of public health/preventive medicine and clinical medicine in an almost parallel manner. We have made efforts: clinical medicine students do have to take several public health courses such as epidemiology and health statistics, but these courses are usually not taken seriously by both student and teacher. Furthermore, the courses on preventive medicine are too theoretical, with insufficient attention to practical hands-on education on the scene or in disease control centers. Students are also far from competent in understanding issues relating health policy, epidemiological information and health management.

Considering this situation, the next step should focus on merging the teaching of clinical and preventive medicines. Teachers from both disciplines should engage more efforts in the education of each other's students, in order to truly broaden the knowledge base of both groups of students and improve their practical skills. There is a long way ahead in implementing this reform.

### Cultivate all types of medical talents with strong professional competency

**GQ Chen**: Clinical or preventive, the major task is to cultivate medical talents with strong professional competency. How are we doing in this aspect?

**MF Li**: I think we should first clarify the connotation and extension of 'professional competency'. Since the beginning of 21st century, professional competency has been highlighted to be the major goal of medical education worldwide. As the COVID-19 pandemic exposed the room for further improvement in medical education, we need

ORIGINAL UNEDITED MANUSCRIPT

to ask: whether there is any overlooked aspect in our understanding of professional competency? For example, did this concept sufficiently emphasize the importance of humanistic and scientific spirit? Should we pay more attention to the competency in dealing with public health knowledge and skills associated with infectious diseases? We should think about these questions and try to better recognize and define the criteria of professional competency.

**H Yan**: Professional competency of the graduates reflects the quality of a medical school. If a medical school has high-quality teachers, facilities and affiliated hospitals, and if it is able to enroll high-quality students and cultivate them with well-designed teaching programs, then the professional competency of its graduates will surely meet the requirements of the society.

In China, we have more than 200 medical schools at all levels, having their own features and aiming for different types of medical workers. We need world-class clinicians, medical scientists and medical educators. We also need grass-root medical workers to serve the primary hospitals in many counties and communities. Actually, the COVID-19 pandemic told us that the grass-root medical workers are crucial for our health care system and we need a lot more of them.

The pandemic tells us the significance of medical education on national security. To strengthen medical education, we should continuously reform the very details of the system: better integration of basic, clinical and preventive medicine, improved course design that encourages case-oriented tutorial discussion, more attention to medical humanistic aspects, more interactions between teachers and students, and strengthening of instruction at all stages of medical education. These are the things we need to do in order to improve the professional competency of our students.

**HB Shen**: While rethinking issues concerning the pandemic, we often mention the cultivation of high-level interdisciplinary medical experts. Firstly, we should help students lay a solid foundation of basic knowledge and skills at their undergraduate stage. With this as the basis, we can further lead them to broaden their perspectives and improve in their abilities of medical research and innovation.

Classified cultivation is a classic institutional design in medical education. Medical schools need to be classified into different types, so that some aim to cultivate high-level experts in specialties, and some focus on training grass-root

ORIGINAL UNEDITED MANUSCRIPT

medical workers. On top of it, students in the same school should also be categorized according to their disciplines and interests, and trained in different categories with distinct curricula. For instance, for those students interested in scientific research with academic pursuit in mind, we can provide them with opportunities and platforms for innovative research training as early as the undergraduate stage. The same goes for graduate students. We should make individual approaches to train different categories of students, establishing different curricula and practice opportunities for each group.

**X Chen**: Maybe we should rephrase the expression of 'cultivate high-level interdisciplinary talents', because it is almost impossible to cultivate this kind of medical talents within five or eight years of education. What possible to cultivate is 'potential high-level interdisciplinary talents'. We hope the medical school graduates can be 'stem-cell-like talents' that have the potential to differentiate into experienced clinical specialists, beloved primary-care physicians, trustable public health experts, or physician-scientists devoted to mechanistic studies of complex diseases.

The primary task of medical schools is to educate the students on how to be a qualified medical worker and how to be an honest and kindhearted human being. An excellent medical worker is not only someone who has superb clinical skills, but also someone brave, diligent, prudent, and fair-hearted. That is what ancient Chinese philosopher Wang Yangming meant by 'the unity of knowledge and action'. We should never forget this point when we are trying to foster the potential high-level compound medical talents.

**M Kuang**: Another question is: what kind of doctors should we cultivate for the future? According to the experiences against COVID-19, tomorrow's physicians should be able to not only cure diseases and but also deal with public health emergencies. Some of them should also be excellent researchers for developing better treatments for multiple diseases. Moreover, besides clinical practitioner and researcher, they should also be good educators for the training of next-generation physicians.

To improve the quality of medical education fundamentally in China, faculty development is a priority. I attend the annual conference of the Association for Medical Education in Europe (AMEE) since 2016 and noticed that around 90% of the participants are clinicians. They consider medical education as the duty of all clinicians, but not just medical school administrators. Physicians themselves should

ORIGINAL UNEDITED MANUSCRIPT

be the major force to improve medical education in China. We should foster more talents who are good at both clinical practice and medical education. Maybe we can set up master's and doctor's programs as part of medical education, and encourage clinicians to learn more about medical education during their continuing education.

**X Chen**: I think the 'the last kilometer' of the running course of teaching is to train qualified medical teachers. In the past 20 years, we have made a great effort to improve the quality of both medical research and clinical practice. But perhaps we should think more about how to give more respect and development space for medical teachers so that they would be glad to devote more effort into teaching and care more about the real development of the students.

### Continuing education after graduation

**MF Li**: To maintain professional competency during one's professional career, all three stages of medical school education, residency training and lifelong continuing medical education, as well as the continuum between these stages should all be reinforced. In the medical school stage, the students should learn enough about public health, preventive medicine and humanistic concerns. In the standardized training of resident physicians, trainees should be able to find their own balance of multi-disciplinary clinical skills. In the continuing education stage, maybe we should think about further strengthening the credit-earning mechanism, based on which a better re-qualification mechanism can be implemented for mid- and advanced-career physicians.

**XH Wan**: In this pandemic, relatively large-scale infection of medical workers occurred in Wuhan Central Hospital and several other hospitals. But the infection rate of respiratory or infectious disease department physicians was relatively low. This implies that most physicians of other departments do not have the experience and skills to protect themselves from serious infectious diseases. Also, at the early stage of the pandemic, many physicians from other departments were not familiar with respiratory diseases and the success rate for emergency treatment of severe patients was relatively low. Considering these problems, I think we should propose more detailed official requirements for teaching practical skills of treating infectious diseases in medical school education.

ORIGINAL UNEDITED MANUSCRIPT

Acute respiratory infectious diseases such as SARS, MERS, COVID-19 and influenza A are highly infectious, rapidly mutating and difficult to control. We should strengthen the education of these diseases so that our future physicians can better treat these patients, control the spread of the viruses and protect themselves.

One good news is that, on March 2nd 2020, the Chinese government issued 16 new occupations and one of them is respiratory therapist. Respiratory therapists are specialists who offer multiple breathing support and airway management therapies to patients suffering respiratory insufficiency. That is what's needed for COVID-19 treatment. In this pandemic, more than 140 respiratory therapists went to Wuhan to help. Now, this occupation is officially recognized and there will be more qualified respiratory therapists and they will be a strong force to deal with future respiratory diseases.

Moreover, I have a suggestion for the continuing medical education. When I was studying in the US in the 1990s, my mentor, a physician and psychiatrist, told me that when AIDS outbreak in the US, all American physicians of all departments have to learn about AIDS for at least two class hours to get the annual credit for their continuing education. I think we should learn from this strategy and require a minimum class hour of new infectious diseases and serious public health events in our continuing medical education.

### More general practitioners are needed

**GQ Chen**: As you have mentioned, we need not only high-level interdisciplinary medical talents, but also general practitioners who can well treat minor diseases, well recognize serious diseases, well transfer the serious patients to superior hospitals, and well manage chronic diseases. General practitioners are critical for our tiered system of medical service and for the rational use of our medical resources. However, we are currently weak in cultivating general practitioners.

**Y Ke**: More than 70% of diseases are age-related chronic diseases. These patients need long-term management by general practitioners in the primary hospital, whose duty is to prevent, treat and long-term follow-up common diseases. On the other hand, the duty of tertiary hospitals is to treat the difficult and severe diseases, to develop and implement new therapies, and to train young medical workers. This tiered system is

ORIGINAL UNEDITED MANUSCRIPT

essential for the treatment of different diseases, but does not mean that physicians in tertiary hospitals are more important than the ones in primary hospitals.

In the past, general practitioners mostly focused on common and chronic diseases, but this pandemic reminds us that they should also play an important role in treating infectious diseases. Most of the mild COVID-19 patients should be diagnosed and treated in primary hospitals. However, without strong primary hospitals, all the patients crowded into tertiary hospitals, occupying the medical resources for severe patients and even triggering further spread of the virus among the crowd. Many patients and their families have to wait anxiously for a long time in the hospital, and the situation of some patients may get worse during this waiting. We do have to promote and accelerate the establishment of a strong primary medical system, starting from the cultivation of general practitioners.

**BR Chi**: The pandemic exposed the problem that we have paid too much attention to specialized medicine education, but are weak in general medicine education. China formally established the college major of general medicine since 2010, as a cross-disciplinary major combining clinical medicine, rehabilitation medicine, preventive medicine and health management. But the educational resource for general medicine remains insufficient, and the educational system has not been standardized or qualified. Now, we have only 2.2 general practitioners for every 10 000 residents. General practitioners is the first line to prevent and control both infectious and non-infectious diseases. We need to increase the population and better educate students majored in general practice with multiple practical skills.

**GQ Chen**: There is a Chinese famous quote that ‘erudition is the premise of becoming a doctor’. To cultivate medical talents - general practitioners or interdisciplinary talents - the most important thing is to cultivate students' ability of active learning. They should not constrain themselves to the courses and tests. They should actively acquire knowledge and skills through all possible ways. That is the premise of cultivating great medical workers.

### Medical education as an important part of the society

**GQ Chen**: Health is the basis of a person and persons are the basis of a society. The pandemic told us again that the medical care system is crucial for people's health,

ORIGINAL UNEDITED MANUSCRIPT

social stability, economic development and national security. It’s important to build a social atmosphere that respects life and medicine.

**H Yan**: The pandemic forces us to reconsider the strategic position of medical education for our society. We hope that the whole society would emphasize more about medical education and the government can invest more in medical education. Moreover, we need a better teacher evaluation system, so that the medical teachers, especially the young teachers would be more inclined to put more effort into the practice and innovation of medical education. That is one of the keys to ensure the quality of medical teachers, and thus, the quality of medical education.

**Y Ke**: Medical education does not exist in isolation. If the medical care system is not well built, it would be impossible for medical schools to attract best students and cultivate high-quality medical workers. So in the one hand, we should call for the society and the government to improve the entire medical system, and on the other hand, we educators should foresee the need of our society and cultivate medical students that are prepared for the future. There are many things that can be done.

**X Chen**: In the coming post-pandemic era, we hope that the society can give medical workers and medical educators more understanding and kindness. Maybe we can establish a national multi-disciplinary medical education committee, which may help to improve the overall quality of medical education.

**MF Li**: In recent years the public conceptually agree that the cost to educate and train a medical professional is high, but do not really recognize the serious consequences that would be caused by medical education deficiencies. In fact, the investment for medical education is still far from enough. Many medical schools have to enroll more students in order to obtain sufficient finance from governments. The COVID-19 pandemic forces us to reconsider this situation. If we do not invest enough on medical education, the consequences may be impossible for our nation to bear.

ORIGINAL UNEDITED MANUSCRIPT

### Cultivate students' responsibility and humanistic spirit

**GQ Chen**: We need social recognition and national investment. But actually, it’s difficult to change the society. All we can do is to change ourselves. It’s always important to improve social responsibility of medical teachers and students.

**YQ Hu**: The 2020 COVID-19 pandemic swept across the globe, impacting almost every country in the world. Tens of thousands of Chinese medical workers resolutely headed to the front-line, successfully saved many lives and controlled the pandemic within a short time. The whole society is greatly touched by the strong social responsibility of the medical workers and began to think about what are good medical workers and how to cultivate qualified medical workers with strong humanistic spirit in the future. We talked much about these questions in our discussion. We should further combine the forces of hospitals and medical schools, and reinforce the connection between medical practice and medical education. We should also emphasize the multi-disciplinary nature of medicine throughout the entire medical education process from medical schools to the graduate medical education and continuing education.

**Y Ke**: There are two major requirements for clinical talents. The first is their ability to cure patients, or the clinical competency. This ability is not reflected by how they are doing in the tests or how much knowledge they have memorized in their minds, but their ability to diagnose and treat complex clinical cases - not only the diseases of a particular discipline, but also infectious disease or other diseases when needed. This ability cannot be obtained only through classroom teaching. So one of the directions of medical education reform is to generally teach the basic concepts and knowledge, and then start practical teaching as early as possible. The students can learn about the detailed medical knowledge, and also develop their abilities to solve practical problems and to communicate with colleagues and patients through the practical teaching process.

The second requirement is the humanistic spirit of medicine. Clinical work requires strong kindness, compassion and empathy. Excellent physicians can well explain the condition of illness to the patients, comfort their anxiety and make up the

ORIGINAL UNEDITED MANUSCRIPT

best treatment plan from the perspective of patients. The best way to foster students' humanistic spirit is through the personal demonstration of the teachers. For example, in this pandemic, the socially responsible behaviors of the elder medical workers greatly influenced many medical students. Besides, it's also important to integrate humanistic spirit into the evaluation criteria of medical workers. Good behaviors should be regularly rewarded.

**MF Li**: The humanistic spirit of medicine includes at least three aspects - the pursuit and uphold of the truth, the willingness and wish to contribute caregiving for human life in need, and the ability to light up human lives with the beauty of human civilization. We should pass the truth, kindness and beauty of medicine on to the next generation through all possible routes, including but not limited to courses, personal demonstrations, and professional environment.

**X Chen**: We all understand the importance of medical humanistic education, but the problem is how to realize it well. In a recent online conference held by the Chinese Ministry of Education, first-line medical educators talked about their experience on how to improve the quality of ideological and political courses. That has some reference values for our future work.

**BR Chi**: I would like to end this panel discussion with the ending of an article published in *Guangming Daily* by Prof. Guoqiang Chen: *The road of medicine is long and obstructed, which needs unremitting efforts and long persistence. The best and only choice for us medical educators should be: never give up, take responsibility and continuously adjust our direction towards the right way.*

### Summary

**GQ Chen**: Many thanks to Prof. Chi and all experts in the discussion. The pandemic manifested the importance of the medical care system. As medical workers, to appropriately react to the big changes that could appear, resembled by the COVID-19 pandemic, we have to clearly recognize and scientifically cope with the situation, and then actively peruse the solutions. Medical education shares fate with our nation and people. It will surely achieve fast development and contribute more to the health of Chinese people and the rejuvenation of China.

ORIGINAL UNEDITED MANUSCRIPT

Li Shao is professor and director of Department of Education of Shanghai Jiao Tong University School of Medicine; Weijie Zhao is an NSR news editor based in Beijing.

#Insert pull quotes

Somewhere in P2

“The entire society … tends to pay more attention to treatment than prevention, resulting in the separation of public health/preventive medicine and clinical medicine in an almost parallel manner.

—Hongbing Shen”

Somewhere in P3

“In China, we have more than 200 medical schools at all levels, having their own features and aiming for different types of medical workers.

—Hong Yan”

Somewhere in P4

“Now, we have only 2.2 general practitioners for every 10 000 residents.

—Baorong Chi”

Somewhere in P5

“The best way to foster students’ humanistic spirit is through the personal demonstration of the teachers.

—Yang Ke”

ORIGINAL UNEDITED MANUSCRIPT
